# A Point-of-Care Ultrasound Rotation for Medical Education Fellows in Emergency Medicine 

**DOI:** 10.24908/pocus.v7i1.14745

**Published:** 2022-04-21

**Authors:** Alanna O'Connell, Al'ai Alvarez, Peter Tomaselli, Arthur Au, Dimitrios Papanagnou, Resa E Lewiss

**Affiliations:** 1 Department of Emergency Medicine, Brookdale University Hospital Brooklyn, NY; 2 School of Medicine, Department of Emergency Medicine, Stanford University Palo Alto, CA; 3 Department of Emergency Medicine, Thomas Jefferson University Hospital Philadelphia, PA

**Keywords:** POCUS, MedEd, fellowships, education theory, procedural training

## Background

A Medical Education (MedEd) fellowship provides emergency medicine (EM) residency graduates the structured and rigorous training to develop skills as educators. Although not accredited by the Accreditation Council for Graduate Medical Education (ACGME), MedEd fellowships have established minimum curriculum standards 1. Our institution’s MedEd fellowship curriculum incorporates an innovative opportunity for fellows: two 3-week rotations in Point-of-Care Ultrasound (POCUS). Here we describe the rationale for using this POCUS rotation to reinforce key MedEd concepts that benefit the MedEd fellows, the POCUS trainees, and the Ultrasound section. Ultimately, we believe this addition in training helps further develop MedEd fellows’ teaching skills, with specific attention to kinesthetic and visual-spatial content.

## Curricular Design

All MedEd fellows graduate from an ACGME accredited EM residency. Consequently, MedEd fellows hold hospital privileges in POCUS 2. That being said, the POCUS training and competency assessment for graduation vary by residency. The POCUS skills of each MedEd fellow also vary. Our institution's rotation began as a proof of concept -- an educational intervention that would provide fellows with the clinical context to develop the ability to teach highly-kinesthetic procedural skills. Although there was anecdotal evidence to suggest this was a valuable educational experience, there was a need to trial the rotation with developmental milestones to capture its impact better 3. We established goals and objectives for each 3-week rotation and outlined experiences within the rotation that would support the stated objectives. We also developed self-directed learning assignments and integrated teaching roles aligned with graduated roles and responsibilities. 

Each MedEd fellow completes two 3-week POCUS rotations over the course of a 12-month fellowship. They attend weekly POCUS section meetings during the rotation, review POCUS images with faculty during the Quality Improvement (QI) segment, and lead journal club discussions. They assist with didactics focused on MedEd theory, employ educational techniques, and conduct competency-based assessments of residents and students. MedEd didactics mostly take the form of a Monday journal club with articles and roundtable discussions centered on a journal article that anchors the conversation around the intersection of POCUS and MedEd. During the rotation, the MedEd fellows organize and teach a weekly scan shift: a bedside POCUS examination and skill day in the Emergency Department for rotation trainees. At this time, they employ education techniques learned over the course of the MedEd fellowship, several of which are described in Table 1. While the applications they teach may be more basic than the advanced applications of a POCUS fellow, they focus on framework, technique, and competency assessments.

## Impact/Effectiveness

### Benefits to the MedEd Fellow

Seven MedEd fellows have completed the POCUS rotations to date. All have shared similar perceived benefits of the rotation. The MedEd fellows found that the scanning shifts provide opportunities to apply MedEd educational models while reinforcing POCUS skills (Table 1). The rotation emphasizes reviewing the specific examination technique immediately before performing it in real-time at the bedside 4. Just-in-time training also serves as a refresher of POCUS content that the MedEd fellows may have learned in residency. Through repetition in performing POCUS, microskills are reinforced, and teaching the application to trainees further allows the fellows to demonstrate progress from novice to a master in skill acquisition 5. The fellows also apply Miller's pyramid^6^ as a competency-based assessment framework to evaluate the resident and medical student learners from “knowing how” to demonstrating a specific POCUS skill. Fellows can also use educational theories, such as cognitive apprenticeship, to frame their procedural teaching 7. Lastly, as an expectation of the POCUS rotation, MedEd fellows are assessed in their technical skills for performing the procedures and demonstrating the ability to teach these skills to other learners. They are also immersed in opportunities to complete workplace-based assessments of learners’ POCUS skills 8. An application of this assessment is using the standardized direct observation tool (SDOT) 9. The POCUS SDOT is designed as an example of a competency-based checklist and provides a snapshot of a resident’s clinical performance that can be repeated longitudinally to document the progression of competency over time. Incorporating learning theories through POCUS theory and skills acquisition, supplemented by bedside hands-on training, provides a framework to expand teaching POCUS skills to teaching other EM procedures, e.g., central venous catheter placements. 

### Benefits to the Ultrasound Fellowship and Section

The EM Ultrasound faculty and fellows have responded positively to the MedEd fellow POCUS rotation. The MedEd fellows contribute to the Monday journal club discussions and provide an evidence-based MedEd perspective to the analysis. EM Ultrasound faculty and fellows learn the education theory behind the POCUS skills they teach thanks to this collaboration. MedEd fellows develop confidence in integrating POCUS into their clinical practice more than other new faculty and fellows. This enhances the EM department's commitment to coding and billing for POCUS diagnostic and procedural examinations. Additionally, as part of the second 3-week rotation, the MedEd fellows can serve as the first reviewer for a portion of the weekly POCUS Quality Improvement (QI) patient examinations. This offers the POCUS section assistance with reviewing all POCUS examinations performed in the ED 10, 11.

**Table 1:** A Framework for MedEd Fellows to Deliberately Practice their Teaching Skills through POCUS.

	**Theory / Model**	**Theory / Model Explained **	**Example in Practice **
**Teaching and Learning**			
	Dreyfus Model of Skill Acquisition 12	Dreyfus describes a stepwise progression for learning specific skills, proceeding through novice, advanced beginner, competent, proficient, and eventually to expert. Levels have anchors in four domains, including components, perspective, decision, and commitment. The time period through which one achieves expert status can last months or years.	The fellow can intentionally choose the POCUS skills to be taught to the learner based on where the learner falls on the Dreyfus model. For novice learners, the fellow can focus on knobology and fundamental principles in probe manipulation. In contrast, for more competent and proficient learners, the fellow can challenge the learner with more advanced ultrasound applications (e.g., examining the vitreous chamber of a patient with acute vision loss). This provides the fellow with the scaffolding to guide learner instruction.
	Miller’s Pyramid of Skill Acquisition 13	Miller walks through several levels of assessment of skills acquisition: knows (demonstrates knowledge), knows how (demonstrates competence), shows how (demonstrates performance), and does (demonstrates action). Depending on the skill, progression may be completed over a variable time period.	The MedEd fellow can apply Miller's Pyramid throughout a shift with a learner for a specific skill. When teaching a learner how to perform a Focused Assessment with Sonography in Trauma (FAST) exam for the first time, the fellow can ask the learner to explain the FAST and its indications and then progress to how it is performed. Throughout the shift, the fellow can prompt the learner to demonstrate the FAST with supervision, and over time perform the FAST independently with remote review by the fellow. This approach prompts the fellow to consider a scaffolded, step-wise approach to teaching a procedural skill.
	Kolb's Experiential Learning Cycle 14	Kolb suggests that learners progress through four separate, but related phases, by which learners experience and process their learning. Phases include: concrete experience, reflective observation, abstract conceptualization, and active experimentation. As phases are non-linear, educational experiences can focus on one phase (i.e., either in series or in parallel).	The MedEd fellow can use the Kolb model to identify ways to better support learning. For learners who prefer concrete experiences, the fellow can identify undifferentiated patients on whom POCUS examinations are performed as a starting point for teaching. For learners who prefer abstract conceptualizations, the fellow can begin instruction by discussing specific scenarios requiring imaging and identifying the best course of action to obtain those POCUS examinations. For learners who prefer active experimentation, the fellow can use a task trainer to practice image acquisition before imaging a patient. And for learners who prefer reflective observation, the fellow can begin a teaching session by asking the learner to reflect on previous experiences with imaging a patient and exploring aspects on previous performance.
**Instruction**			
	Just-in-Time Training 15	Web-based assignments designed to complete before giving and receiving instruction on POCUS examinations.	The fellow can prompt the learner to review the necessary steps to successfully perform POCUS-guided fascia iliaca nerve block in a patient with a hip fracture immediately preceding the procedure. This may take the form of watching a FOAMed video and/or verbally describing critical steps of the procedure with the learner.
	Microskills Teaching	Specific and discrete actions that can be observed and repeatedly practiced into understandable and repeatable skills.	When performing a cardiac POCUS examination, the fellow can break down the procedure into its steps and specifically teach those steps. For example, position the patient, drape the patient, and manipulate the phased-array ultrasound probe for a subxiphoid view.
	Hands-on Bedside Teaching	Perform POCUS procedure, interpret images, integrate into patient care.	The fellow instructs trainees on Heart/Lung/Inferior Vena Cava (IVC) POCUS examinations in a patient with undifferentiated dyspnea and periodically steps-in to optimize image acquisition and/or quality. This may be in the form of literal hands-on assistance or verbal coaching during the scan.
**Assessment of Learners**			
	Standardized Direct Observational Assessment Tool (SDOT) [6]	Checklist of steps to complete the POCUS examination.	The fellow can use the SDOT to assess students and residents during a scanning shift.
	Direct Observation	The trainer observes the trainee performing the assessment and assesses the ability to perform it accurately and properly.	Fellows can be prompted to directly observe the learners' POCUS skills. They would be prompted to not interfere with scanning or image acquisition and only observe skills, which would be referenced during the debriefing that follows the scan.
	Quality Improvement	A systematic approach to the analysis of practice performance and efforts to improve performance.	During Quality Improvement sessions with ultrasound section faculty, fellows can provide feedback on recent ultrasound studies performed in the ED for clinical care. They learn to give remote feedback to the clinicians on improving future ultrasound examinations, such as optimizing depth and gain or re-educating on how to differentiate the IVC from the aorta.

## Limitations

There are several limitations we would highlight. The benefits are anecdotal and have not yet been studied using rigorous program evaluation methods. The efficiency and confidence that the MedEd fellows report after the POCUS rotation have not been measured. Future studies should evaluate the impact of the curricular addition of POCUS to the MedEd Fellowship program objectives, the trainees, the POCUS section, and the department. 

## Conclusions

The POCUS rotation for MedEd fellows provides an opportunity for fellows to develop the skills and confidence in POCUS while applying the MedEd theories they are studying. MedEd fellowship programs may consider the addition of a POCUS rotation to their core curriculum to help meet program learning objectives. 

## Disclosures

DP was a 2020 Macy Faculty Scholar through the Josiah Macy Jr. Foundation.

REL serves on the medical advisory board of Echonous.
